# Falling into a deep dark hole: Tongan people’s perceptions of being at risk of developing type 2 diabetes

**DOI:** 10.1111/hex.13056

**Published:** 2020-05-22

**Authors:** Julienne Faletau, Vili Nosa, Rosie Dobson, Maryann Heather, Judith McCool

**Affiliations:** ^1^ Epidemiology and Biostatistics Section Faculty of Medical and Health Sciences School of Population Health The University of Auckland Auckland New Zealand; ^2^ Pacific Health Section Faculty of Medical and Health Sciences School of Population Health The University of Auckland Auckland New Zealand

**Keywords:** Pacific Island, prediabetes, risk perception, Tonga, Type 2 Diabetes

## Abstract

**Background:**

Prediabetes is a precursor for type 2 diabetes. Compared to the New Zealand/European and other population groups (24.6%), the prevalence of prediabetes is higher within Pacific groups (29.8%). The diagnosis of prediabetes presents a potential opportunity to intervene to prevent progression to type 2 diabetes.

**Objective:**

To develop an understanding of how being ‘at risk’ of developing type 2 diabetes is perceived by Tongan people with prediabetes living in Auckland, New Zealand.

**Methods:**

The Kakala and Talanga Tongan methodologies underpinned this study. Twelve one‐on‐one, semi‐structured interviews with Tongan patients who had prediabetes from a primary health‐care clinic in Auckland, New Zealand, were conducted. Thematic analysis was used to identify recurrent themes from the data.

**Results:**

Participants were not aware of their prediabetes diagnosis, emotions associated with the diagnosis reflected fear and disbelief and a perception of imminent danger. Family history informed perceptions of the risk of developing type 2 diabetes. Participants could not differentiate prediabetes from type 2 diabetes, and recollections of being ‘back in the Islands’ of Tonga were consistent with healthy lifestyles.

**Conclusions:**

Prediabetes appeared to be poorly understood and was believed to be irreversible, which could discourage behaviour change, social and physical improvements in health. Appropriate culturally tailored messages to accompany a prediabetes diagnosis, including cause and management, would be beneficial for Pacific peoples.

## 1 BACKGROUND

Over 200 000 people in New Zealand (NZ) have been diagnosed with diabetes, specifically type 2 diabetes (T2D).^1^ This is a possible underestimate of the actual burden of disease due to under‐diagnosis.^1^, ^2^ Compared to the NZ/European and other population group (24.6%), the prevalence of prediabetes is higher within Pacific groups (29.8%).^2^ Pacific people make up 7% of the total NZ population^3^ with Tongan people comprising 20.4% of Pacific peoples in NZ.^4^ Pacific peoples are at high risk of T2D both within their homelands and as migrants to NZ.^5^ Overall, Pacific populations in NZ are more likely to have a non‐communicable disease, compared to NZ/European people.^5^, ^6^, ^7^

Prediabetes described by the World Health Organization is a state of higher than normal high blood sugars (41‐49 mmol/mol) but not high enough to meet the diagnostic criteria for T2D (>50 mmol/mol).^8^ The chronic and non‐acute nature of prediabetes can lead to late diagnosis and delayed treatment. Established risk factors associated with prediabetes include ethnicity (particularly for ethnic groups in NZ, being of Māori, Pacific Islander or South Asian descent)^5^ and increasing age. Other risk factors include a sedentary lifestyle, poor nutrition (and overweight), high blood pressure and family history of T2D.^9^ Although prediabetes can lead to T2D, it can be managed through lifestyle changes such as diet and physical activity.^9^

The diagnosis of prediabetes presents a potential opportunity to intervene to prevent progression to T2D. The challenge of engaging with people who have been diagnosed with prediabetes is that they are likely to feel well, therefore may see little need to change their behaviours.^10^ This ambivalence may reflect a pervasive belief that T2D is largely pre‐determined, an inevitable consequence of ageing or genetics.^11^ People who are aware that they are at risk for developing T2D have the best chance of preventing the onset of T2D by changing their diet and increasing their physical activity.^12^ Maintaining good health while being at risk for a chronic condition is argued to be contingent on access to credible information on practical options to manage risk.^11^ Yet, people who live with an elevated risk profile are often least likely to have access to and/or engage with health information and system support.

The NZ health system is designed to deliver quality, accessible health care to a diverse population.^13^ Despite an intention to be accessible, the majority of information for supporting people with long‐term conditions, such as diabetes, is via didactic and Western biomedical health information approaches, for example pamphlets or in‐person consultations explaining the importance of dietary changes. Research suggests that access to credible, culturally relevant health information is essential, but not the only element required to support behaviour change.^14^ Misinterpretation of prediabetes information between patient and clinician during a consultation could lead to inaccurate knowledge about the definition, diagnosis and treatment.^15^, ^16^, ^17^, ^18^, ^19^ Any misunderstanding of what prediabetes is and being at risk of developing T2D may affect the likelihood of initiating any necessary behaviour changes such as changing diet or increasing physical activity.^18^ Some argue that the risk needs to be communicated effectively to mobilize behaviour change.^19^, ^20^, ^21^

An individual's perception of being ‘at risk’ of developing a life‐threatening disease inevitably shapes how they think and feel and what they do. ^21^ The literature pertaining to risk perception of individuals at high risk of developing T2D and those diagnosed with prediabetes highlights several key points. Firstly, there are inherent differences in knowledge and understanding of risk and prediabetes between clinician and patient.^15^, ^21^, ^22^ Often, health‐care providers base diagnoses on physiological markers and use medical terminology that patients do not necessarily understand, whereas a patient's perspective of being at risk is based on illness perception,^23^ social factors such as culture, family history and present lifestyle, their emotional state and ability to understand medical information at that point in time.^16^, ^17^ Studies find that promoting patient‐centred dialogue about prediabetes is important because it ensures that patients can comprehend risk on a good level of understanding to make decisions for their health.^19^ What constitutes patient‐centred information, of course, is variable. Secondly, other studies found that patients who were at risk of developing T2D were unaware of their prediabetes diagnosis^24^ leading to guilt, distress and fear.^15^, ^18^, ^21^ However, when the diagnosis was made known, patients felt that this was not serious because prediabetes was asymptomatic.^18^ This led to ambiguity about the meaning and seriousness of the disease. Thirdly, other studies reported that patients with prediabetes acknowledged that family history of T2D directly impacted their understanding of being at risk of developing T2D.^18^, ^25^ One study revealed that having a family history of T2D prevented positive behaviour change in patients due to belief in a pre‐determined outcome of T2D.^25^ On the other hand, a diagnosis of prediabetes motivated and encouraged other patients to change their eating habits and increase physical activity.^25^ Those who were at high risk of developing T2D were more likely to draw upon the negative experiences of family members who lived with T2D to initiate positive lifestyle changes for themselves. The adverse experiences of seeing family members take diabetes medication to manage their condition and avoid limb amputations and subsequently death were actually detrimental actions to prevent T2D. It is evident that patients at high risk of developing T2D comprehend prediabetes in multiple ways. Though it is almost always based on family history of T2D, this is an interesting proposition that despite strong familial connections, personal risk of developing T2D might be an opportunity to mobilize collective or personal action to change.

This study was designed to examine how Pacific people, specifically Tongan people with prediabetes, understand and conceptualize the concept of risk as it pertains to T2D. Pacific people experience a higher risk of developing T2D; understanding perceptions of risk may provide clues for future behaviour and social group‐level change interventions.

## 2 METHODS

### 2.1 Pacific qualitative methodologies

This study used the Kakala^26^ and Talanga^27^ Tongan methodologies. The Kakala methodology is the process of garland making in the Tongan context. There are three stages in this process: 1. Toli: to pick flowers needed to make the garland, 2. Tui: to weave the flowers in a respectable fashion, and 3. Luva: to present the garland to the intended wearer. Each stage of the Kakala methodology is equivalent to the research process of recruiting participants, data analysis and dissemination of findings.^28^ The Talanga is the manner of interactive talking with a purpose between people.^27^ These methodologies were essential to adhere to during the research process for cultural consistency, respectful sharing of experiences and authentic dialogue.

### 2.2 Recruitment

Eligibility criteria required participants to be of Tongan descent and with a clinical record of being diagnosed with prediabetes (HbA1c between 41 and 49 mmol/mol) with no subsequent T2D diagnosis. Participants were purposively sampled from a Pacific primary health‐care clinic located in South Auckland, NZ. The health coach who is responsible for managing recall for people with diabetes is the prediabetes educator in the clinic assisted with recruitment. The health coach provided information about the study to all eligible patients about the study. Those who were interested in participating in an interview contacted the clinic to arrange a convenient day, and time for the interview was scheduled.

### 2.3 Interview procedure

All interviews were conducted between August and November 2018. The first author (JF) conducted interviews in a private room at the health clinic. The face‐to‐face Talanga began unrecorded with a brief introduction in Tongan by the researcher. Interviews were conducted interchangeably in English and Tongan based on participants’ preference. Discussions began with questions about the participant's knowledge and understanding of T2D, family history of T2D and their understanding of being what being ‘at risk' of developing T2D meant to them. Interviews were digitally recorded and lasted up to 40 minutes.

### 2.4 Transcribing and Translating

Recorded interviews were transcribed verbatim in the language it was conducted (eg Tongan or English). All transcripts in Tongan were translated to English for analysis. The first author completed the translation process. The transcripts were then reviewed by an independent Tongan scholar to ensure that the English translations aligned with the Tongan transcript version.

### 2.5 Data analysis

Transcripts were de‐identified, imported into and managed using NVivo version 12. Braun and Clarke's six‐stage thematic analysis method was used to elicit critical themes.^29^ The first author became familiar with the data by reading and re‐reading the printed hard‐copy transcripts. The transcripts were imported to NVivo for further analysis. Using NVivo, the textual data were coded inductively, line‐by‐line generating several codes. A codebook was generated to detail key concepts. The codebook was reviewed by the research team to refine specific themes and ensure consistency of understanding. Codes that were similar in meaning were grouped into a theme. Key themes were identified by their significance, based on the codes. A thematic map was then created to illustrate connections and defined the key themes.

## 3 RESULTS

### 3.1 Participant characteristics

A total of twelve patients who have recently been diagnosed with prediabetes were interviewed. Table 1 presents the demographic characteristic of the sample. Most participants were female (nine), and three participants had other comorbidities, including stroke, hepatitis B and muscular dystrophy. Eleven participants were born in Tonga, and all eleven had a family history of T2D.

**Table 1:** Demographic information of participants

Characteristic	n
Gender	
Female	9
Male	3
Age (y)	
20‐29	1
30‐39	3
40‐49	3
50‐59	5
Family history of T2D	
Yes	11
No	1
Health issues (prediabetes +)	
Gestational diabetes	2
Stroke	1
Hepatitis B	1
Muscular dystrophy	1
Place of birth	
Tonga	11
New Zealand	1
Work status	
Unemployed	4
Employed	8
Education	
Secondary (College level)	7
Tertiary (University level)	5

### 3.2 Themes

There were four key themes from the interviews: (a) no awareness of prediabetes diagnosis, (b) emotions associated with prediabetes diagnosis, (c) lack of differentiation between prediabetes, being at risk and T2D, and (d) ‘back in the Islands’ of Tonga was consistent with a healthy lifestyle. Each theme is described below with supporting quotes.

#### 3.2.1 No awareness of diagnosis of prediabetes

It was noted from the outset that participants were mostly uninformed about their personal risk of developing T2D. They were made aware of their diagnosis of having prediabetes during initial recruitment (phone calls) and also reminded upon their arrival to the study interview. Participants were shocked and expressed their worry of not knowing their health condition.

…at risk of diabetes, I didn't think I had, or I was at risk of developing diabetes, and I had no knowledge of it. (Male, 53 years)

I was thinking oh wow I didn't even know that they didn't even let me know that I was in that border…if I am at risk and I’m worried at the at risk (Female, 38 years)

All participants expressed that the primary reason for attending the clinic at any time was to seek medical treatment for an unwell child or themselves if they had symptoms of the ‘flu’. It was evident that participants did not see the need to be screened for prediabetes, T2D, or to carry out an HbA1c test when visiting their health‐care provider.

I only come when I have a sickness or the cold or flu, or whatever, and then that's when I'll do a blood test. But I don't come just to test my diabetes. (Male, 45 years)

Three female participants described that they felt uncomfortable in terms of being heavy—concerning their weight and health in general. They worried that the uncomfortable feeling they had meant might have developed T2D. Their initial reaction was to have a blood test to check their sugar levels.

This is the first time that they've told me but it's alright because I, I had a feeling because this year, it's a very uncomfortable for me, uncomfortable year for me with my weight and my health and stuff I can always feel it in my, you know and I've always feared that I will maybe, and I get it so yeah. (Female, 40a years)

I wanted to do the test, I kind of felt I'm nearly having diabetes. I hope I'm not (Female, 52 years)

#### 3.2.2 Emotions associated with a prediabetes diagnosis

The common emotions associated with views of being at risk and having a prediabetes diagnosis were disbelief and perceptions of being in danger and fear.

### 3.3 Disbelief

With minimal awareness of their prediabetes diagnosis prior to the interview, participants expressed disbelief when told of their elevated risk of developing T2D, questioning the results they had received from the health coach. One participant did not think it could ever happen to her because she did not have a family history of T2D.

I didn’t think it would ever be me and so when she said it was just over the normal range, I was like ‘oh my goodness’ and so that was really scary. (Female, 26 years)

A few female participants did not seek medical help since they did not see or feel physical illness despite being overweight. Being overweight was not perceived as a health issue needing medical attention as it is not always accompanied with other health concerns. Despite being overweight, the women reported feeling fine; therefore, the prediabetes diagnosis was unexpected.

…you know my heart nearly stopped cause I never thought, I keep telling my sisters… ‘My big size is okay because I don't have any sickness or illness. You know my eating at night is okay because I don't have any sickness or illness’…I'm shocked, and I have to do something…so that I can be safe from this disease. (Female, 53 years)

### 3.4 Perception of being in danger

All participants expressed that being given a diagnosis of prediabetes (and therefore at an elevated risk of developing T2D) was feeling like they were in a state of danger, reinforced with a warning. Most participants described prediabetes as a sign of being on the verge of hitting T2D. Being at risk of developing T2D was metaphorically likened to taking a wrong step and ‘falling into a deep dark hole’. In other words, the diagnosis was like the first step along a dangerous path that metaphorically speaking requires an individual to walk with caution.

Being at risk means to me…I think it’s, its’ danger. You’re about to, you know, hit diabetes. (Female, 40b years)

To me, at risk is being in a position of danger. When you say that you're at risk, it is when you are at a step of nearly falling down a deep dark hole. (Female, 53 years)

### 3.5 Fear

Participants experienced the effects of T2D vicariously through seeing family members live with and eventually die from the disease. This familial experience played an important role in both shaping and exacerbating the fear associated with a prediabetes diagnosis.

It's like a feeling of fear because as time goes on it's like I will get it. (Female, 52 years)

To be honest, fear. I was scared to, at the time I got the result when it said that I've got diabetes. (Female, 35 years)

When discussing the implications of prediabetes developing into T2D, more than half of the participants expressed that they feared T2D because they did not want to have insulin injections and commented that they knew of family members whose limbs were amputated as a result.

I don't want to have to always like inject insulin or yeah just don't want that lifestyle. (Female, 26 years)

yeah my dad’s brothers, two of them, they were diabetic, I just remembered, yes, even up to a point where they got their leg amputated, even my grandmother her leg got amputated too. (Female, 53 years)

Several participants expressed concern and fear because they recognized the damaging effects T2D had on their grandparents, parents and siblings. For most participants, they were the sole dependent person in their family to monitor their parent's food intake, sugar levels and especially their medication.

…you know my mum got diabetes…make sure she eats, make sure she takes her tablets, you know, they have less sugar stuff. (Female, 40b years)

…my husband had diabetes. The disease is very dangerous. It’s a possibility you could die from it…it was very had to look after him. If I don’t look after his insulin, I notice that he gets sick. (Female, 59 years)

All eleven participants with a family history of T2D spoke about death in their family due to T2D. It was a health issue that seemed normalized within Tongan families, especially identifying the generations before them that passed away due to T2D and its complications.

…on my dad’s side, all of them died because of diabetes…my dad’s sisters have all died… and they've got brothers that have died, and it's thought it's because of their kidneys, but that's the result of the diabetes. (Female, 35 years)

The majority of the participants were worried about the consequences of developing T2D for the future of their family and children. They expressed fear for their young children and who would look after them if they developed T2D. Participants recognized that they needed to be in good health to take care of their family members in the long run.

…because if it happens, if I get sick, who will stay and look after our son and things like that…If we are both alive and be in good health, good health, then there will be nothing to worry about. If we both get sick, there will be a lot of problems, differences in how we live, especially within our little family. (Female, 38 years)

#### 3.5.1 Understanding prediabetes

Participants understanding of prediabetes was constructed upon experiences of family history of T2D. As a result, a few participants could not distinguish a diagnosis of prediabetes from T2D. One male participant expressed that being at risk was clearly having T2D and not being able to return to good health from the diagnosis.

At risk and already reach it, you know, just about the same… only one step ahead then you can get there and then I don’t know if you gonna get back to that, from that. (Male, 56 years)

The use of the term at risk or prediabetes did not hold any significance to the participants of possible opportunities to change their trajectory because they viewed prediabetes (a precursor) as fundamentally having T2D.

Nearly having diabetes, is like reaching it, or you've already got the diabetes. (Female, 40a years)

One particular participant expressed that because she was diagnosed with prediabetes, she would consider herself to have T2D. On the outset, this notion was expressed in a positive light as motivation to change her poor dietary behaviour and lifestyle.

I say that I’ve got diabetes so that I can try and finish the diabetes by looking after myself. At risk, I hate that word. I will just say I am part of those people with diabetes…so that I look after myself, in what I eat, my health. (Female, 59 years)

#### 3.5.2 ‘Back in the Islands’ of Tonga

Eleven of the twelve participants recalled their upbringing in Tonga where they perceived themselves to be relatively healthy. They described the way of life as ‘simple’, consuming locally grown produce, including taro leaves and spinach during the weekdays. A predominately vegetarian diet was standard for many families; meat was an exception on Sundays.

In Tonga, we almost always have green leaves. Sometimes we don't have portions of beef and we you think about it we only eat lamb on Sundays. Yes, and that was the normal. We just eat spinach during the week, put coconut cream and cook the food; we were full. We were full, and we didn't feel too big. (Female, 35 years)

Treats such as fizzy (soda) drinks, chocolate biscuits and potato chips were rare luxuries for the participants who grew up in Tonga. Participants described drinking coconuts and water rather than fizzy. Their mode of transportation and range included walking to and from school, church and the city town centre. Some participants recalled working as farmers on their land growing crops to feed their families. These daily activities constituted their exercise routine.

When I grow up back home…we were drinking rainy water, and if you want to have a sweet drink then we have the niu mata that coconut…I remember those days, we never, we hardly drink any sweet drinks because not only we couldn’t afford it…I think the most important thing is back in those days we walk everywhere. We walk to school, we walk back home, we walk to church, but we didn’t know that’s our exercising. (Male, 56 years)

Although they expressed that they did not have any spare money, participants appeared satisfied with what they could eat, drink and live on in Tonga. Tongan breakfast foods such as topai (dumplings made out of flour, sugar, coconut water and coconut shreds) for breakfast was enough, easy to make and healthy.

…we have less money, but we got bread, we got topai, heuheu for breakfast. When we go to school, dad always give us $2 $1 to buy bread and lemonade that's our lunch, or we buy, or we go and have ahh pawpaw at school. (Female, 53 years)

Participants described consuming fruits at school and eating locally grown foods. The reference to ‘back in the Islands’ of Tonga reflected the importance of traditional foods and living a healthy lifestyle from former days living in the Kingdom of Tonga.

## 4 DISCUSSION

This study found that participants’ awareness of prediabetes and their personal risk of developing T2D were minimal. Several expressed disbelief at their status of having prediabetes, yet many had familial associations with T2D, which may account for their expressions of fear. Participants could not differentiate between prediabetes and being at risk for T2D highlighting that the terms ‘being at risk’ and ‘prediabetes’ require more explanation, that is both clinically accurate and culturally appropriate for better understanding for Tongan patients. Recollections of being ‘back in the Islands’ in Tonga were consistent with personal ideals of ‘good health’.

Our findings suggest that respondents’ awareness of their own prediabetes status and the broader understanding of prediabetes as well as their personal risk of developing T2D were minimal. This aligns with previous research highlighting challenges associated with a condition that is predominantly asymptomatic (elevated HbA1c levels) until T2D is advanced.^24^, ^30^, ^31^, ^32^ The NZ health system offers screening for prediabetes to adults over 25 years of age with heart disease, obesity, family history of T2D and women with a history of gestational diabetes.^33^ Primary health‐care providers may perform opportunistic screening as an attempt to capture those at high risk of developing T2D.^34^, ^35^ Some argue that health outcomes are a reflection on patient engagement with the health system. Bean, Cundy and Petrie^23^ reported that in order to encourage positive behaviour change in Pacific communities, health‐care providers must understand Pacific cultural practices and perceptions of T2D. By encouraging primary health‐care clinicians to communicate screening outcomes to patients with prediabetes in a culturally appropriate way has the potential to make patients aware of their own risk and what they can do to mitigate the onset of T2D.

Participants responded to the knowledge of being at risk of developing T2D, with disbelief and fear. This finding concurs with previous research^23^ which found that Pacific peoples expressed high emotional distress towards T2D, in particular, Tongan people because they were more likely to experience or be familiar with T2D complications.^36^ Tongan adults interviewed in the present study referred to being at risk of developing T2D as a danger or ‘falling into a deep dark hole’. This metaphor suggests a perception that T2D is an abyss—place of irreversible loss. In another study, participants viewed prediabetes as ‘being on a train that they could not stop’.^37^ Strachan and colleagues found that for many, it was inevitable that the progression of T2D will occur, regardless of health support since the risk factors associated with prediabetes were primarily embedded in the participant's everyday lives. However, studies report that prediabetes is reversible with lifestyle modifications,^18^, ^38^ including evidence that lifestyle interventions could lead to a 27% reduction of diabetes incidence over 15 years.^12^ Lifestyle modifications have been shown to delay the development of T2D by three to four years,^39^ and in patients who exercised, and with every kilogram in weight loss, the risk of T2D has been found to reduce by 16%.^40^ In addition to structural and environmental factors, health literacy and tailored health promotion initiatives can be instrumental in reducing risks associated with T2D.^41^ The prediabetes screening appointment presents an opportunity to actively engage patients with practical, realistic and culturally appropriate lifestyle adaptions to stave off T2D.^42^ The importance of culturally relevant health literacy and education tools should not be underestimated in communicating important information.

Understandably, many participants held a poor understanding of the difference between prediabetes and T2D. This may be due to several factors, including the relatively new and possibly ambiguous term ‘prediabetes’ as well as asymptomatic nature of both prediabetes and T2D.^43^ Studies found that patients who were diagnosed with prediabetes did not see the seriousness of the prediabetes to make immediate changes.^15^ It is essential, therefore, that patients with prediabetes know that the condition can lead to T2D and that with changes to diet and physical activity, the risks can be reduced.^43^ The findings of our study reflect concern that there is no simple Tongan word for prediabetes or term to describe being at risk of developing T2D. In this instance, risk and prediabetes were described as a concept accompanied with a description that was relatable to Tongan cultural understanding of illness. Herein again lies a problem for both clinicians and patients in communicating risk and prediabetes diagnosis. To alleviate misunderstanding between clinical and lay medical terms,^24^ the imbalance of knowledge between clinicians and patients^44^ and the impact of poor health literacy on T2D risk and selfcare,^42^ clear and relevant communication strategies for patients with prediabetes are vital. Delivering key messages about prediabetes that is accessible and culturally appropriate is imperative for any behavioural changes to be implemented.

Interestingly, recollections of being back in the Islands of Tonga denoted an optimistic view that the lifestyle in Tonga contributed to good health. Evidence shows that returning to traditional lifestyles and foods may be protective against some non‐communicable disease such as T2D.^45^ A study by Shintani, Hughes, Beckham and O'connor^46^ found that when twenty native Hawaiian's resumed to a traditional Hawaiian diet for twenty‐one days, a 7.8 kilograms in weight loss was experienced. They found that the consumption of a Western diet, rather than overeating, was the cause of obesity and consequently T2D.^46^ O’Dea reported similar outcomes with Australian Aboriginal people who had T2D.^47^ Participants went on the traditional Aboriginal diet for seven weeks. As a result, participants lost an average of eight kilograms in weight.^47^ There is therefore potential that reverting to or increasing traditional foods in everyday diets and lifestyles incorporating greater physical activity could potentially reduce the risk of developing T2D. However, the challenge associated with the rise of globalization worldwide creating obesogenic environments still poses difficulties and challenges for Pacific peoples in NZ and the Pacific region.y^48^

A strength of this study includes conducting interviews in both Tongan and English languages. The use of Pacific methodologies to underpin the research design ensured that Pacific worldviews were reflective of the participants. The generalizability of these findings, however, cannot be applied to all Pacific peoples in NZ or the Tongan people living in the Kingdom of Tonga. This study was conducted in a Pacific primary health‐care provider located in an area with a large enrolled Pacific population which allowed for purposive recruitment of participants. Due to the small number of participants, and the sampling method, the perspectives are not generalizable. It is a limitation that recruitment was from one clinic and there is potential that the way prediabetes is communicated in this clinic is not reflective of other clinics. The sample was limited to people who were engaged with the health system and had a diagnosis, and therefore, the findings are not a reflection of the perceptions of those who are managing their prediabetes, and those not engaged with the health system.

## 5 CONCLUSIONS

To improve awareness of a prediabetes diagnosis within Tongan populations, primary health‐care screening and appropriate communication of being at risk of developing T2D are vital. Developing feasible, culturally relevant ways of communicating about prevention and management of prediabetes and T2D within the Tongan community is essential to support social and behaviour change.

## ETHICS

This study received approval from The University of Auckland Human Participants Ethics Committee on the 2nd of June 2017 for three years (reference 019242). All participants provided informed consent before taking part.

## CONFLICT OF INTEREST

All authors have no conflict of interest to declare.

## Data Availability

Data available on request from the authors. The data that support the findings of this study are available from the corresponding author upon reasonable request.
